# The genetic effect and molecular function of the SOCS5 in the prognosis of esophageal squamous cell carcinoma

**DOI:** 10.7150/jca.51806

**Published:** 2021-02-22

**Authors:** Pei-Wen Yang, Ya-Han Chang, Li-Fan Wong, Ching-Ching Lin, Pei-Ming Huang, Min-Shu Hsieh, Jang-Ming Lee

**Affiliations:** 1 Department of Surgery, National Taiwan University Hospital & National Taiwan University College of Medicine.; 2 Graduate Institute of Pathology, College of Medicine, National Taiwan University, Taipei, Taiwan.

**Keywords:** esophageal cancer, esophageal squamous cell carcinoma (ESCC), suppressor of cytokine signaling-5 (SOCS5), single nucleotide polymorphisms (SNPs), epidermal growth factor receptor (EGFR)

## Abstract

Expression of cytokines and growth factors have been shown to be highly correlated with the prognosis of esophageal squamous cell carcinoma (ESCC), a deadly disease with poor prognosis. The suppressor of cytokine signaling (SOCS) family of proteins are key factors in regulating cytokines and growth factors. Yet the role of the SOCS proteins in ESCC is hardly investigated. We currently investigated the prognostic role of SOCS5 in ESCC. We analyzed the prognostic effects of 16 single nucleotide polymorphisms (SNPs) within the SOCS genes in 632 ESCC patients. We repeatedly observed that the 3 SNPs in SOCS5, SOCS5:rs3814039, SOCS5:rs3738890, and SOCS5: rs3768720, were significantly correlated with both overall (OS) and progression-free survival (PFS) of ESCC patients (rs3814039, p=0.032 for OS and p=0.009 for PFS; rs3738890, p=0.016 for OS, and p=0.008 for PFS; rs3768720, p=0.005 for OS and p=0.002 for PFS). SOCS5: rs3768720 was also significantly associated with distant metastasis (Ptrend=0.028).

The luciferase assay revealed that SOCS5:rs3814039 and SOCS5: rs3768720 might influence the prognosis by regulating SOCS5 expression. Functional analysis demonstrated SOCS5 was able to regulate epidermal growth factor receptor (EGFR) expression and migration activity of ESCC cells. Furthermore, Patients with strong SOCS5 in normal tissues exhibited significantly better PFS (P=0.049) and reduced risk of distant metastasis (P=0.004) compared to those with weak SOCS5 expression. Overall, our study demonstrates the novel function of SOCS5 in ESCC prognosis. The genetic polymorphisms and expression of SOCS5 could serve as a novel therapeutic biomarker for improving the prognosis of ESCC.

## Introduction

Esophageal squamous cell carcinoma (ESCC) is the major cell type of primary esophageal cancer, accounting for about 90% of the disease worldwide, and is highly correlated with environmental factors 1-2. As no efficient targeted therapeutic agent has been discovered, the standard treatment for locally advanced esophageal cancer is preoperative (neoadjuvant) concurrent chemoradiotherapy (CCRT) combined with surgical dissection 3. Esophageal cancer patients encounter high risk of either local recurrence or distant metastasis after treatment 4-6. The 5-year survival rate of patients with esophageal cancer is less 20% under multiple treatment modalities 7.

Cytokine-derived signaling has been extensively observed to dominate adverse clinical outcomes in ESCC patients 8. Increased levels of IL-6 9-11, IL-1 12, IL-8 13, IL-12 and IL-18 14 have been reported in the plasma in ESCC patients and correlated to poor prognosis or adverse clinical (or pathological) changes in ESCC. Growth factors represent another important group of signaling molecules that play crucial roles in ESCC progression and prognosis. Vascular endothelial growth factors (VEGFs) are frequently reported to be over-expressed in ESCC and correlated with tumor growth and poor clinical outcome 8. Other growth factors, e.g. hepatocyte growth factor (HGF) 15 and insulin growth factor-II (IGF-II) 16, have also been reported to be expressed at increased levels in ESCC patients and to correlate with poor survival. Our previous study found that epidermal growth factor (EGF) and VEGF were highly correlated with the prognosis of ESCC 17. Thus, cytokines and growth factors have frequently been identified as important biomarkers in ESCC patients.

The family of suppressor of cytokine signaling (SOCS) proteins represents one of the key mechanisms regulating signaling derived from cytokines and growth factors 18, and plays important anti-inflammatory and tumor suppressive roles. The family contains 8 members, SOCS1, SOCS2, SOCS3, SOCS4, SOCS5, SOCS6, SOCS7 and CISH, each containing 3 domains, a less conserved N-terminal domain, a classic Src-homology 2 (SH2) domain, and a highly conserved SOCS box at the C-terminus 18-19. The SOCS box domain consists of a cul box and BC box and mediates ubiquitination and proteasome-dependent degradation of target proteins. Degradation of receptors or associated proteins is one of the mechanisms by which SOCS proteins negatively regulate the signaling of cytokine or growth factors.

Hypermethylation of SOCS1 and SOCS3 has also been observed in esophageal adenocarcinoma (Barrett's adenocarcinoma) 20 and in ESCC 21. A recent study reported that SOCS1 gene therapy induced an antitumor effect in an ESCC xenograft mice model 22. Studies of the genetic polymorphisms in the SOCS family of genes in solid tumor are not extensive. Single nucleotide polymorphisms (SNPs) of the SOCS1 and SOCS3 genes have been analyzed in colorectal cancer patients 23-24. However, no association of these SNPs and cancer development has been found.

SOCS5 is known to be a negative regulator of epidermal growth factor (EGFR) signaling 25-26. It has been shown that SOCS5 is induced by EGF and that negative feedback regulates the expression of EGFR by proteasome dependent degradation in Chinese hamster ovary (CHO) cells 26. The function of SOCS5 SNPs has hardly been investigated. One study demonstrated that 6 of the SNPs of SOCS5 have no significant correlation with type 1 diabetes mellitus 27.

Little is known about the role of the SOCS family of proteins in ESCC prognosis. We currently investigate the clinical relevance of genetic polymorphisms of the SOCS family and the possible prognostic function of the SOCS5 protein in ESCC.

## Materials and methods

### Study population

The study was performed retrospectively and approved by the ethical committee of National Taiwan University Hospital (NTUH, 201412172RINB). A total of 632 patients were enrolled in NTUH during 2000 to 2013. The inclusion criterion was diagnosis with primary ESCC, and the exclusion criteria were inability to give informed consent, pregnancy, and pediatric patients. The demographics and clinical information of the patients were obtained from the Tumor Registry of NTUH and/or medical chart-review. Ten milliliters of whole blood was obtained from patients before treatment and stored in a -80 °C freezer. Overall survival duration (OS) was defined as the duration between surgical dissection of esophageal cancer or initial diagnosis in patients who did not undergo tumor dissection and mortality of the patient. Progression-free survival (PFS) was considered as the interval between surgical dissection or initial diagnosis of the disease and detection of local recurrence, tumor progression or death.

### DNA extraction and genotyping

Genomic DNA was isolated from the buffycoat containing peripheral blood mononuclear cells (PBMCs) using the QIAamp DNA kit (Qiagen, Hamburg Germany) according to the manufacturer's instructions. The genotypes of 16 SNPs within the SOCS family of genes were analyzed using MassARRAY® iPLEX Gold technology according to the manufacturer's instructions (Agena Bioscience, San Diego, USA) as described previously 28.

### Cell cultures

CE81T/VGH and KYSE-70 are human ESCC cell lines derived respectively from a Taiwanese patient 29-30 and a Japanese patient 31, and were cultured in DMED/F12 and RPMI complete medium respectively. Het-1A is a SV40 T-antigen immortalized human esophageal epithelial cell line 32, and was cultured on CellBind dishes (Corning) in BEGM Bullet kit medium (Lonza). HEsEpiC (Human Esophageal Epithelial Cells) a non-tumorigenic esophageal cells, was purchased from ScienCell Research Laboratories and was cultured in EpiCM-2 (ScienCell) medium. HEK293T (or simply 293T) is a cell line derived from the human embryonic kidney HEK293 cell line and harboring a mutant SV40 large T antigen, and were cultured in RPMI complete medium. A549 (human non-small cell lung cancer), ECV304 (human bladder carcinoma), and HeLa cells (human cervical cancer) were grown in RPMI and DMEM (HeLa) complete medium respectively. All the cells were cultured at 37^o^C incubator containing 5% CO_2_.

### Protein extraction and western blotting

Tissue protein from ESCC patients was extracted with super lysis buffer (containing 3% SDS, 2 M urea, and 2% 2-mercaptoethanol) 33. Total protein from the cell lysate was extracted using RIPA buffer (150 mM NaCl, 50 mM Tris-HCl [pH 7.5], 1% Igepal-CA630, 0.5% sodium deoxycholate, 0.1% SDS, 50 mM NaF, 1 mM Na3VaO4, and complete protease inhibitor cocktail). Various amounts of protein were mixed with SDS-PAGE sample buffer and resolved by SDS- polyacrylamide gel electrophoresis (SDS-PAGE) followed by Ponceau S staining (Sigma) and western blotting analysis with specific antibodies as described previously 34. The primary antibodies used for protein detection were anti-SOCS5 (ab97283, Abcam, for endogenous SOCS5 detection), anti-SOCS5 (Santa Cruz, for GFP-SOCS5 detection), anti- EGFR (C74B9, Cell Signaling), anti-Myc (for myc-EGFR detection, 9E10, Millipore), anti-HER2 (Cell Signaling Technology, CST), anti-phospho-AXL (CST), anti-AXL (Abcam), and anti-β-actin (clone 4, Millipore) antibodies. The signal intensities values were determined by ImageQuant 5.1 (Molecular Dynamics, Inc.).

### Plasmid construction

The myc-DDK-tagged SOCS5/pCMV6 (myc-SOCS5/pCMV6) expression plasmids were purchased from OriGene Technologies (RC206267). The open reading frame of SOCS5 from myc-SOCS5/pCMV6 was subcloned into the SgfI and MluI sites of the pCMV6-AN GFP vector (PS10019, OriGene) to generate GFP-fused SOCS5 plasmid (GFP-SOCS5/pCMV6-AN-GFP). The promoter region containing SOCS5: rs3814039_G and SOCS5: rs3814039_C were PCR amplified by PCR using specific primer pairs (forward, 5'- CCGCTCGA GTGCAGGCGTGAACTATGCTT -3'; reverse, 5'- GGAAGATCTGCCTACCGT GACCAATAGCA -3') to generate a fragment of around 730 base pairs (bps) and cloned into the pGL4.17 [*luc2*/Neo] vector (Promega). The template for PCR amplification was buffy coat DNA from an ESCC patient carrying the heterogeneous genotypes CG at rs3814039. The clones containing rs3814039_G (SOCS5:rs3814039_G/pGL4.17) or rs3814039_C (SOCS5:rs3814039_C/pGL4.17) were confirmed by sequence analysis.

To analyze whether SOCS5:rs3768720 modulates SOCS5 expression, we constructed SOCS5: rs3768720_C/pmirGLO and SOCS5: rs3768720_A/pmirGLO reporter plasmids at the XhoI and Pme I sites of the pmirGLO vector (promega), a dual-luciferase miRNA target expression vector, for luciferase assay. The DNA fragments containing SOCS5: rs3768720_C (A) were PCR amplified using the reverse primer 5'-CCGCTCGAGACACCTGTAGCTCTATCCGC-3' paired with the forward primers 5'-GGGTTTAAA CCTCCGGTCCCCA AAGG TTG-3 or 5'-GGGTTTAAACCTCCGGTCCCCAAAG GGTG-3' to generate SOCS5: rs3768720_A or SOCS5: rs3768720_C respectively. The clones designed as SOCS5: rs3768720_C/pmirGLO or SOCS5: rs3768720_A/pmirGLO were confirmed by sequence analysis.

### Transient transfection and MG132 treatment

To transiently over express GFP-SOCS5 and myc-tagged EGFR (myc-EGFR) in HEK293T or ESCC cells, the cells were seeded overnight and transfected with indicated amounts of GFP-SOCS5/ pCMV6-AN-GFP and/or pcDNA6A-EGFR plasmids, a kind gift from prof. Mien-Chie Hung 35, using TurboFect™ (Origene) according to manufacturer's instructions. At the indicated time post-transfection, the cells were harvested for protein extraction and functional assay. For the proteasome inhibitor treatment, the cells were treated with 0 (solvent control), 1, and 10 µM of MG132 (Sigma) at 24 hours post-transfection and incubated for 24 hours.

### Luciferase assay

For the promoter activity assay, the cells were co-transfected with the proper amount of SOCS5: rs3814039_G/pGL4.17 or SOCS5: rs3814039_C/pGL4.17 together with pRL-SV40 Renilla luciferase reporter (Promega, for internal control) using TurboFect™ (Origene) at the basal level or induced by EGF or IL-6. At 48 hours post-transfection, both firefly and Renilla luciferase activity were determined by the Dual luciferase assay system (Promega) following the manufacturer's instructions. For the assay of miRNA target expression, the ESCC cells were transiently transfected with SOCS5: rs3768720_C/pmirGLO or SOCS5: rs3768720_A/pmirGLO using TurboFect™. Firefly and Renilla luciferase activity were both analyzed as described above.

### Wound healing assay

The procedures of the wound-healing assay (*in vitro* scratch assay) are mostly based on previous studies 36-37. CE81T cells were cultured in DMEM/F12 medium containing 5% FBS in 6-well. After overnight incubation, the cells were transfected with indicated amount of GFP-SOCS5 expression plasmid (0, 0.5, 1, 2, and 3 µg per well) using Lipofectamine 2000 (Thermo Fishers Scientific) followed by replacement of fresh medium containing 2% FBS. The cells were trypsinized and re-seeded into the culture-insert 3 well to create cell-free gaps at 24 hours post transfection. After another 24 hours incubation, the cells were pre-treated with 10 µg/ml of mitomycin C (Sigma) in serum-free medium for 2 hours followed by the removal of culture insert. The remaining cells were maintained in fresh serum-free medium. More than 8 images of the scratch were obtained without overlapping between images at indicated time points by a light microscope system (Nikon ECLIPSE TS100). The rates of wound healing were analyzed by image J software.

### Statistical analysis

Demographic and clinical characteristics of ESCC patients by survival status in training, replication, and combined groups were compared by a Pearson chi-square test or Fisher exact test. The hazard ratios (HRs) of death and disease progression for the genotypes of the SOCS SNPs adjusted for other potential covariates were determined by multivariate Cox regression analysis. Binary logistic regression was applied for the odd ratios (ORs) of local recurrence and distant metastasis for the genotypes of the SOCS SNPs adjusted for potential covariates. Crude survival curves, both for overall and for progression-free survival of patients with each genotype, were constructed by the Kaplan-Meier method and compared statistically by the log-rank test. A two-sided *p*-value equal or less than 0.05 was considered statistically significant. All statistical analyses were conducted using SPSS version 17.0 (SPSS Inc., Chicago, IL).

## Results

We first investigated the clinical relevance of the single nucleotide polymorphisms (SNPs) of the SOCS family of genes, including 5 SNPs of SOCS1, 3 SNPs of SOCS3, 5 SNPs of SOCS5, and 3 SNPs of CISH, which were selected based on a previous literature search 27, 38-40. A total of 632 patients diagnosed with ESCC were enrolled in the study and randomly assigned to a training set (n=268) or replication set (n=364). Among these subjects, 583 patients (92.2%) were male, 406 patients (64.2%) received esophagectomy, and 446 patients (70.6%) were treated with CCRT. There were significant differences in the distributions of esophagectomy and CCRT treatments by survival status in the combined group (P<0.001 and P=0.006 respectively for survival, Table 1). As expected, T-stage, N-stage, and M-stage were also strongly significantly associated with survival status (P<0.001). Age, gender and tumor site were borderline significantly associated with survival status in the combined group (P=0.087, P=0.080, and P=0.058 for age, gender and tumor site respectively, Table 1).

The germline genotypes of these SNPs were analyzed and correlated with overall survival (Table 2) or progression-free survival (Table 3) by multivariate Cox regression analysis. In the training group (n=268), 8 SNPs, including 2 in SOCS1 (rs33932899 and rs243324), 1 in SOCS3 (rs2280148), 3 in SOCS5 (rs3814039, rs 3738890, and rs3768720) and 2 in CISH (rs2239751 and rs622502), were significantly or borderline significantly correlated with overall (OS) or progression-free survival (PFS) (Table 2 and Table 3). In an independent replication group (n=364), the correlation was repeated only in the 3 SNPs of SOCS5 (rs3814039, p=0.127 for OS and p=0.034 for PFS; rs3738890, p=0.119 for OS and p=0.051 for PFS; rs3768720, p=0.092 for OS and p=0.016 for PFS; Table 2 and Table 3). The prognostic relevance of the 3 SNPs was statistically significant for both OS and PFS in the combined group, which combined the subjects in the training and replication sets (rs3814039, CC vs. GG, HR [95% CI]= 1.35 [1.03-1.74], p=0.032 for OS, HR [95% CI]=1.40 [1.09-1.79], p=0.009 for PFS; rs3738890, GG vs. CC, HR [95% CI]=1.38 [1.06-1.80], p=0.016 for OS, HR [95% CI]=1.41 [1.09-1.81], p=0.008 for PFS; rs3768720, CC vs. AA, HR [95% CI]=1.46 [1.12-1.91], p=0.005 for OS, HR [95% CI]=1.51 [1.17-1.95], p=0.002 for PFS; Table 2 and Table 3). Furthermore, patients carrying the C allele of SOCS5: rs3768720 had about a 2-fold increased risk of distant metastasis (CA vs. AA, OR [95% CI]= 1.96 [1.09-3.52], p=0.025; CC vs. AA, OR [95% CI]= 2.17 [1.08-4.40], p=0.030, Table 4). None of these 3 SNPs had an obvious effect on local recurrence among the ESCC patients.

**Figure 1:**
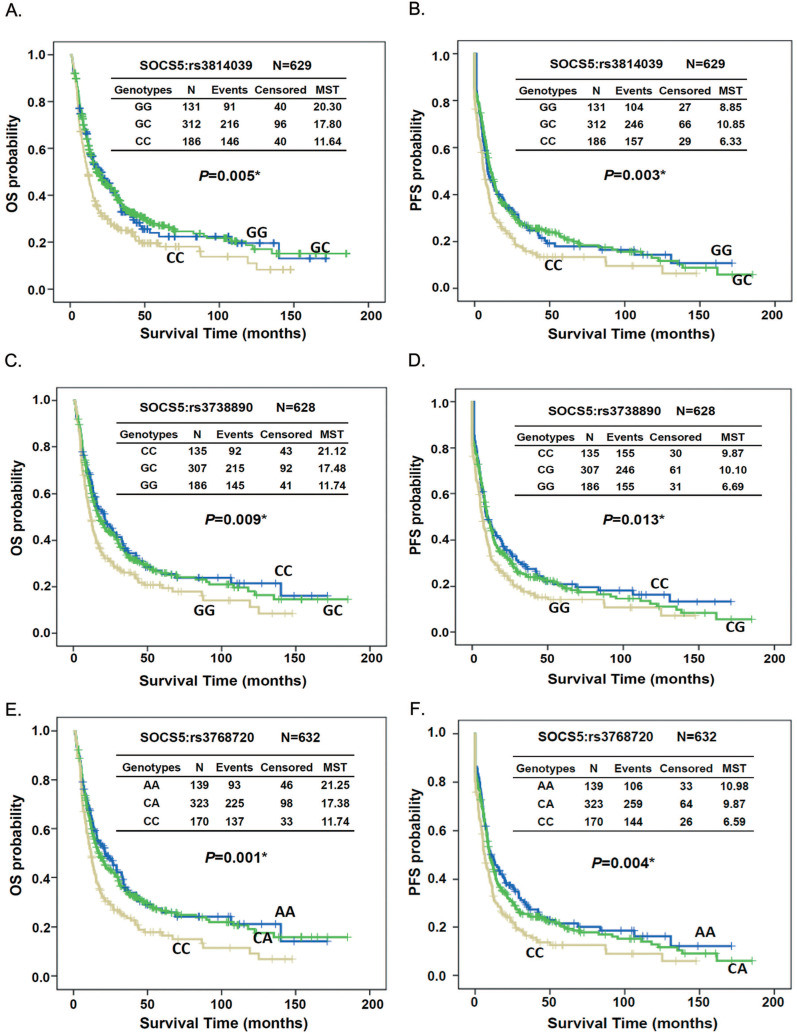
Kaplan-Meier estimates of overall survival (OS, A, C, and E) or progression-free survival (PFS, B, D, and F) by the genotypes of SOCS5:rs3814039 (A and B), SOCS5: rs3738890 (C and D), and SOCS5: rs3768720 (E and F) among total ESCC patients. MST: median survival time (months).

Kaplan-Meier survival analysis demonstrated both OS and PFS differed significantly between patients with different genotypes of SOCS5:rs3814039, SOCS5: rs3738890, and SOCS5: rs3768720 when tested among all subjects (rs3814039, log-rank P=0.005 for OS, Fig. 1A, MST 6.33 vs. 8.85 months, log-rank P=0.003 for PFS, Fig. 1B; rs3738890, GG vs. CC, MST 11.74 vs. 21.12 months, log-rank P=0.009 for OS, Fig. 1C, MST 6.69 vs. 9.87 months, log-rank P=0.013 for PFS, Fig. 1D; rs3768720, CC vs. AA, MST 11.74 vs. 21.25 months, log-rank P=0.001 for OS, Fig. 1E, MST 6.59 vs. 10.98 months, log-rank P=0.004 for PFS, Fig. 1F). Notably, the prognostic effects of these 3 SNPs were more evident in patients who did not undergo esophagectomy, both for the OS and PFS (Fig. S1 and Fig. S2).

**Figure 2:**
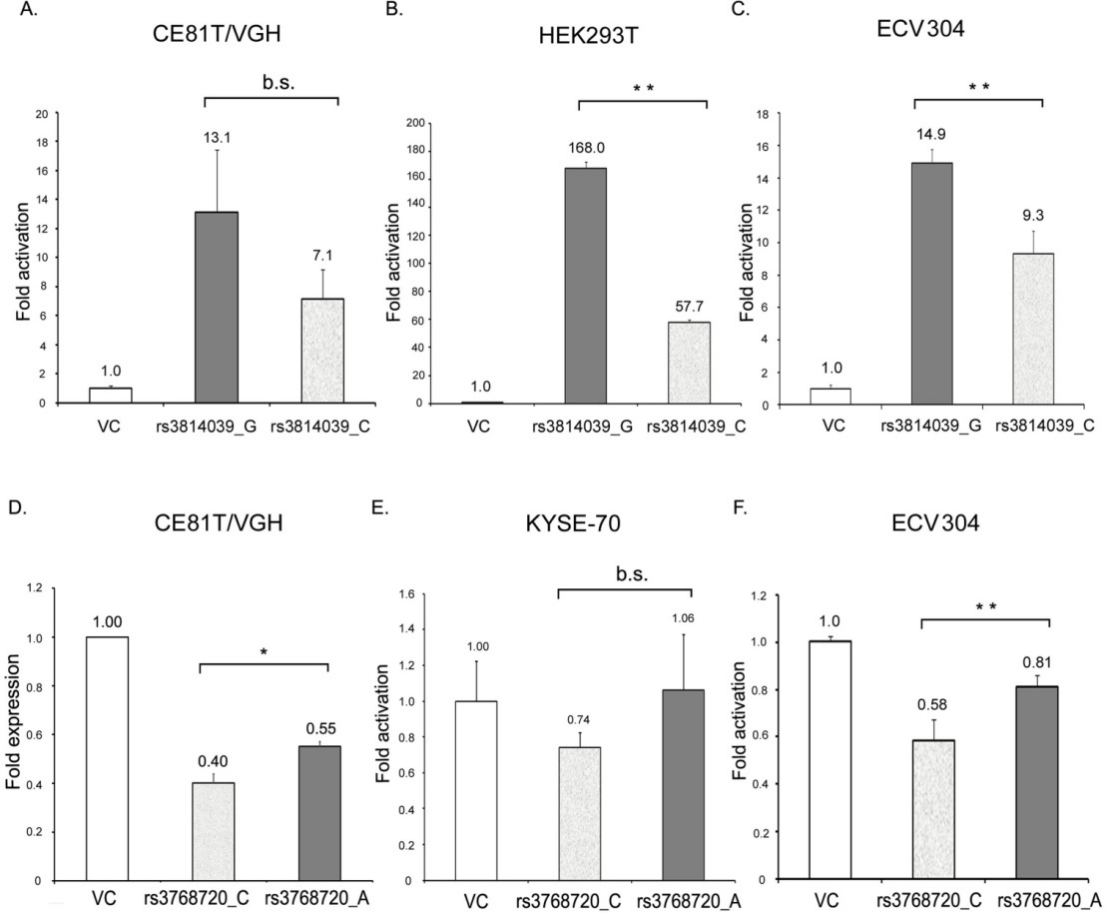
The effect of SOCS5:rs3814039_C/G and SOCS5:rs3768720_C/A on the gene expression of SOCS5 was analyzed by luciferase reporter assay. (A-C) Relative promoter activity of SOCS5 5'UTR (5'-untranslated region) harboring rs3814039_C or rs3814039_G in CE81T/VGH ESCC cells (A) HEK293T cells (B), and ECV304 bladder carcinoma cells (C). (D-F) Relative reporter gene expression of pmirGLO vector harboring SOCS5: rs3768720_C or SOCS5: rs3768720_A in CE81T/VGH (D) or KYSE-70 (E) ESCC cells, and ECV304 bladder carcinoma cells (F). The reporter activity was determined by the luciferase activity and normalized by renilla luciferase activity in each reaction. Fold change in activation (or expression) represents the ratio of reporter activity relative to the vector control. Similar results were repeatedly obtained 3 to 5 times. *, 0.01<P<0.05; **, P<0.01, b.s., borderline significance (0.05<P<0.10); n.s., not significantly different (P> 0.10).

SOCS5:rs3738890 is an intronic variant of uncertain significance. SOCS5:rs3814039 is within the promoter region of SOCS5 while SOCS5: rs3768720 is within 3'UTR (untranslated region) of the SOCS5 gene. Both of the SNPs may play regulatory roles. To clarify whether SOCS5:rs3814039 and SOCS5: rs3768720 influence gene expression, we constructed reporter plasmids containing C (rs3814039_C) and G (rs3814039_G) alleles of rs3814039 within the promoter region of SOCS5 for reporter assay. We transiently expressed vector (VC), rs3814039_G and rs3814039_C reporters in CE81T ESCC cells. The promoter region containing the unfavorable C allele (rs3814039_C) exhibited a reduced activity compared to that harboring the G allele (rs3814039_G) with borderline significance (P=0.094, Fig. 2A). A similar trend was observed in HEK293T cells (Fig. 2B), and human bladder carcinoma ECV-304 cells (Fig. 2C). We then investigated whether SOCS5:rs3768720 modulates SOCS5 expression. We successfully constructed SOCS5: rs3768720_C and SOCS5: rs3768720_A in pmirGLO, a dual-luciferase miRNA target expression vector. As expected, the reporter carrying the unfavorable allele C exhibited reduced expression of the reporter gene compared to the reporter carrying the favorable allele A in CE81T ESCC cells (Fig. 2D). Decrease expression was also observed in C-carried reporter in KYSE-70 cells with a borderline significant trend (P=0.093, Fig. 2E). Strong significant reduction of rs3768720_C was observed in ECV-304 cells (Fig. 2F). These results suggest that both SOCS5:rs3814039 and SOCS5:rs3768720 might influence ESCC prognosis mediated by modulating SOCS5 expression.

**Figure 3:**
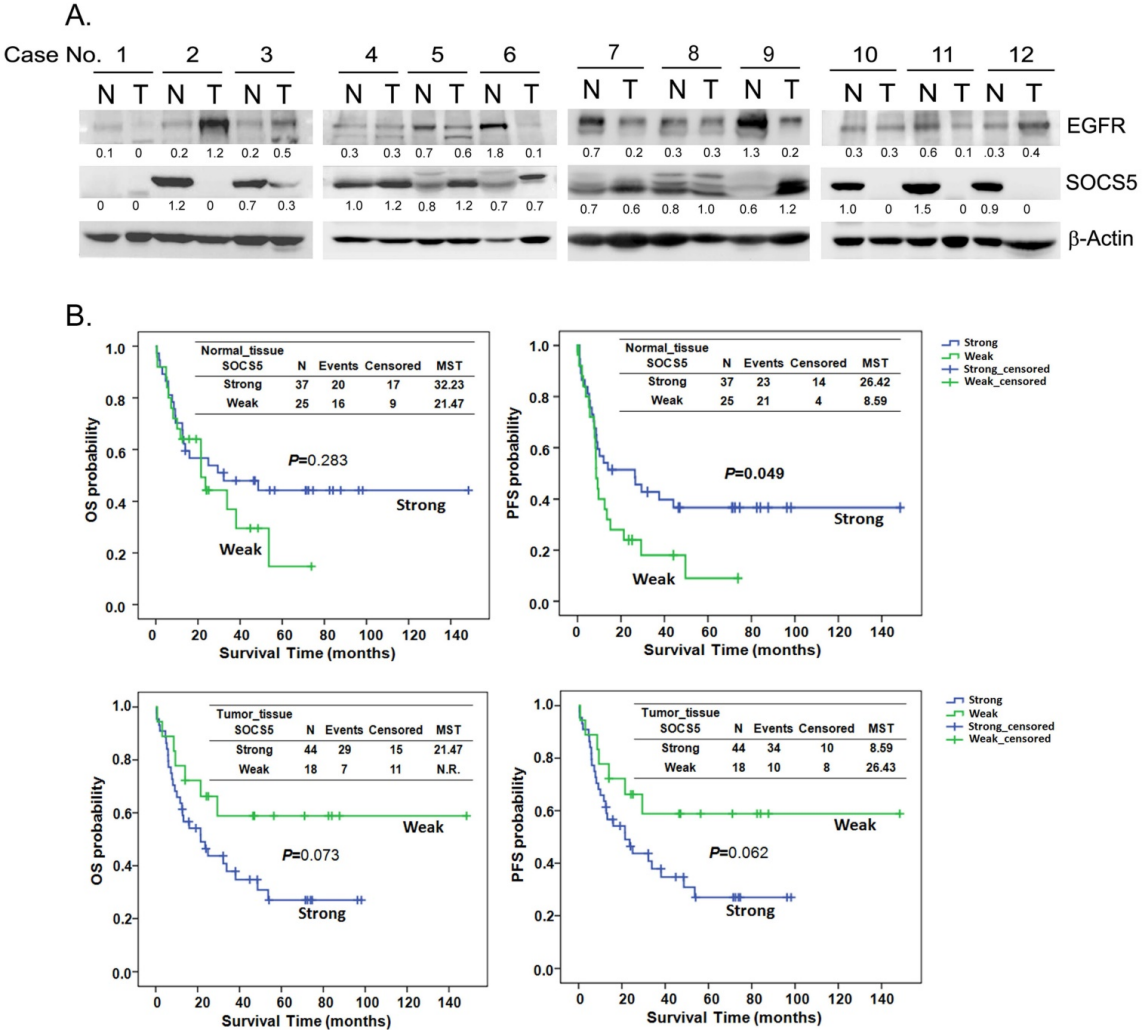
Correlation of EGFR and SOCS5 expression in esophageal tissue of patients with ESCC. (A) Representative expression of SOCS5 and EGFR in esophageal tissues. Total protein was extracted from non-cancerous (normal, N) and tumorous (T) esophageal tissue samples. The expression of EGFR and SOCS5 were analyzed by western blotting using anti-EGFR and anti-SOCS5 antibodies respectively. β-actin was used as a loading control. The ratio of intensities normalized to loading control are indicated below the signals. (B) The expression levels of SOCS5 in the tissues were sub-grouped into strong or weak (normalized ratio less than 20%) groups based on the ratio of intensities which normalized to loading control (β-actin or Ponceau S staining) 58. Kaplan-Meier estimates of overall survival (OS) or progression-free survival (PFS) by the expression level or the expression change of SOCS5 in normal (N) or tumor (T) tissue. MST, median survival time; N.R., the median survival was not reached.

To investigate the expression correlation between SOCS5 and EGFR, we detected the expression of SOCS5 and EGFR in 62 sets of adjacent normal and tumor tissues by western blot. Multiple species of endogenous SOCS5 could be detected with the molecular weight around 55 to 60 KDa (KiloDalton) both in normal and tumor tissues. The results from 12 sets of tissue are displayed in Fig. 3A. Weak expression (including no expression) of SOCS5 was noted in 25 normal (25/62, 40.3%) and in 18 tumor tissue samples (18/62, 29.0%). Interestingly, there was significantly different trend of SOCS5 expression between normal and tumor tissues in most of the tissue sets (35/62, 56.5%). Among these sets, 21 (21/62, 33.9%) and 14 (14/62, 22.6%) tumor tissue samples displayed up-regulation and down-regulation of SOCS5 respectively compared to their corresponding normal tissue samples. EGFR was frequently detected in the sets of esophageal tissue (Fig. 3A). Notably, the difference in expression between SOCS5 and EGFR of tumor tissue was negatively correlated with that of corresponding normal tissue in about half of the tissue sets (30/62, 48.4%) which revealed the possible negative regulating role of SOCS5 in EGFR expression.

Patients were sub-grouped into the strong-expression and weak-expression groups based on the expression of SOCS5 in esophageal tissues. There was no significant difference between the demographic and clinical variables of the 2 groups (revised Table S1). The Kaplan-Meier estimates revealed a trend of better OS among patients with strong SOCS5 expression in normal tissue compared to those with weak expression (P=0.283, MST =32.23 vs. 21.47 in the strong and weak groups respectively, Fig. 3B). However, the trend associated with tumor tissue was just the opposite. Strong SOCS5 in tumor tissue was correlated with worse OS (P=0.073, MST =21.47 vs. N.R. [not reach] in the strong and weak groups respectively, Fig. 3B). Furthermore, weak expression of SOCS5 in normal, but not in tumor tissue, was strongly associated with distant metastasis after surgery (P=0.004, Table 5). Over half of the patients (58.3%) with weak SOCS5 expression had distant metastasis in contrast to the patients with strong expression of which only 22.2% had distant metastasis (Table 5).

**Figure 4:**
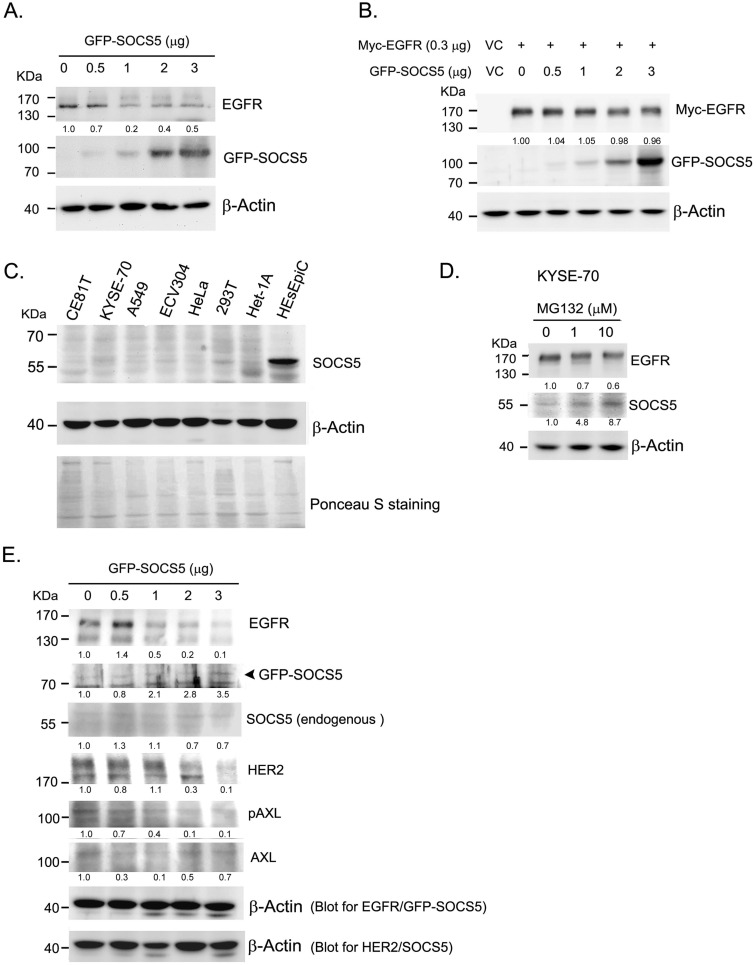
Function of SOCS5 in regulating EGFR expression in cell model. (A)(B) Regulation function of SOCS5 in cells. Indicated amounts of GFP- tagged SOCS5 (GFP-SOCS5, 0 to 3 μg) were transiently expression (A) or co-expressed with myc-tagged EGFR (Myc-EGFR) (B) in 293T cells for 48 hours followed by protein extraction and detection. (C) The basal expression of SOCS5 were detected from different types of cells, including CE81T, KYSE-70, A549, ECV304, HeLa, 293T, Het-1A, and HEsEpic cells. Ponceau S staining was served as loading control. (D) KYSE-70 ESCC cells were treated with 0, 1, and 10 μM of MG132 for 24 hours. Expressions of EGFR and SOCS5 were detected by western blotting using specific antibodies. (E) Increasing amounts of GFP-SOCS5 (0 to 3 μg) were transiently transfected into Het-1A cells. Protein expression profiles were analyzed at 48 hours post-transfection. Both endogenous SOCS5 and GFP-SOCS5 were detected by using anti-SOCS5 antibody. β-actin served as a loading control. The ratio of normalized intensities relative to control (lane 1) are indicated below the protein signal.

To further analyzed whether SOCS5 was able to regulate EGFR expression, we transiently over-expressed GFP-tagged SOCS5 (GFP-SOCS5) in 293T cells. GPF-SOCS5 dose-dependently down-regulated endogenous EGFR in 239 T cells (Fig. 4A). However, the regulatory effect was not obviously on exogenous myc-tagged EGFR (Myc-EGFR) (Fig. 4B). We further analyzed the expression of SOCS5 in a few cell lines. SOCS5 was hardly detected in cancer cells, including ESCC (CE81T and KYSE-70), A549 (lung cancer), ECV304 (bladder cancer), and HeLa (cervical cancer) cells. Notably, SOCS5 could be markedly detected in non-tumorigenic esophageal squamous epithelial cells, HEsEpiC cells (Fig. 4C). The SOCS family of proteins have been found to be rapid turnover short-lived proteins whose degradation is mediated by the proteasome-dependent pathway 41-42. Since the transcript of SOCS5 could be detectable in ESCC cells (data not shown), we hypothesized that SOCS5 protein might be degraded in ESCC cells. As expected, treatment with the proteasome inhibitor MG132 markedly increased the expression of endogenous SOCS5 (Fig. 4D). Thus, SOCS5 expression might be regulated by proteasome-dependent degradation in ESCC cells.

We cannot perform functional assessment in ESCC cells since both endogenous or exogenous GFP-SOCS5 were hardly detected in these cells. Het-1A is a transformed non-tumorigenic esophageal cells which expressed low level of SOCS5. Transiently over-expressing GFP-SOCS5 was likely to be detected in Het-1A cells (Fig. 4E). EGFR and HER2 were significantly decreased with increased amounts of GFP-SOCS5 transfection. The phospho-AXL (pAXL) was also decreased with increased expression of GFP-SOCS5 (Fig. 4E).

**Figure 5:**
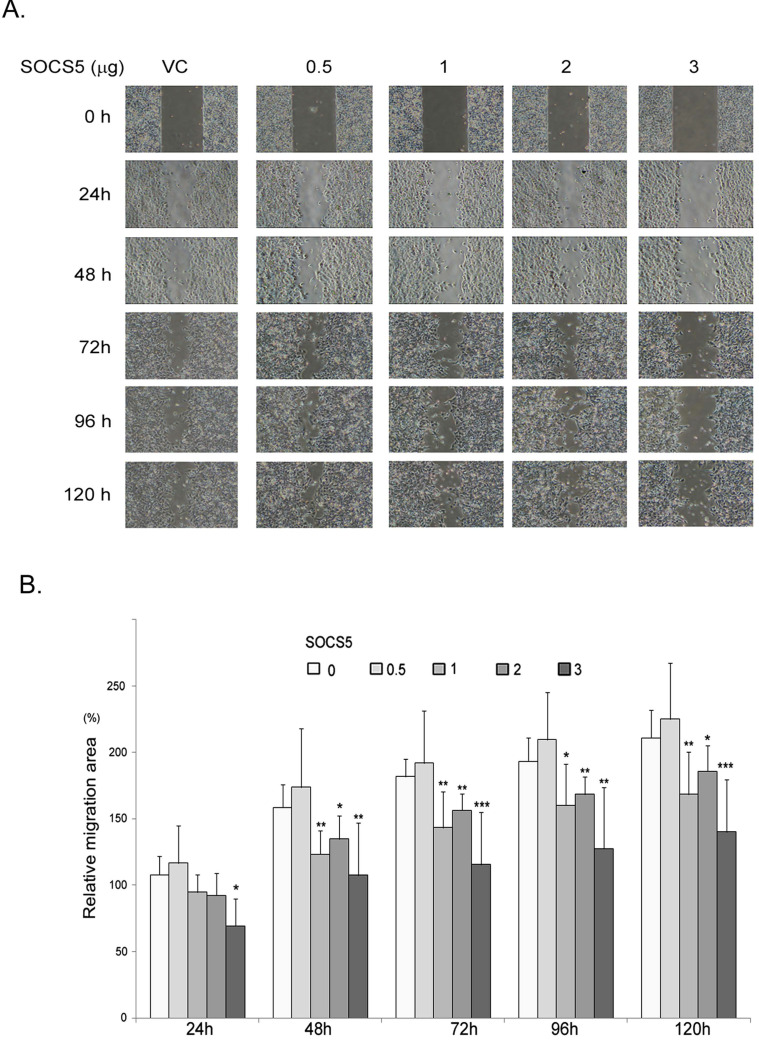
Inhibition of cell migration by SOCS5. Control vector or indicated amount of GFP-SOCS5 (0.5, 1, 2, and 3 μg/well) was transiently expressed in CE81T cells for 24 hours followed by wound-healing assay. (A) Representative images of wound length of CE81T cells expressing indicated amounts of GFP-SOCS5 at 0, 24, 48, 72, 96. and 120 hours. (B) Relative migration area of the ESCC cells transfected with indicated amounts of GFP-SOCS5. Graphs represent the average calculated scratch area ± SD (N=8) (*, P<0.05; **, P<0.01; ***, P<0.001).

Because SOCS5 seems to be correlated with distant metastasis of esophageal cancer (Table 5), we finally investigated the effects of SOCS5 on cell migration by wound-healing assay. We transiently over-expressed increased amounts of GFP-SOCS5 in low-serum cultured ESCC cells and observed that high expression of SOCS5 (3 µg/well) obviously suppressed the migration activity of ESCC (Fig. 5A). The relative migration area are markedly decreased in cells transfected with higher amounts of GFP-SOCS5 (1, 2, and 3 µg/well) at 48, 72, 96, and 120 hours post scratch (Fig. 5B).

## Discussion

Regarding the function of the SOCS family in regulating the expression of cytokine and growth factors, we hypothesized that the prognostic function of certain SOCS proteins might be observed in ESCC. The correlation between cancer prognosis and the SNPs of SOCS genes has not been widely investigated. It has been reported that a SNP in the SOCS1 gene (rs243327) correlated with the response to imatinib treatment in newly diagnosed chronic-phase chronic myeloid leukemia 43. By screening the prognostic effect of the SNPs of the SOCS genes, we identified one family member, SOCS5, as a potential prognostic factor for ESCC. Our study clearly demonstrated the novel effect of 3 SNPs of SOCS5, rs3814039, rs3738890, and rs3768720, on the survival of ESCC patients (Table 2, Table 3 and Fig. 1). Patients carrying unfavorable genotypes of these SNPs had significantly less median overall or progression-free survival time compared to those with favorable genotypes (Fig. 1), especially in patients who did not undergo esophagectomy (Fig. S1 and S2). Patients who did not undergo surgery were usually those who were inoperable. Those with metastatic disease. Unfavorable genotypes of SOCS5 might play roles such as the promotion of metastasis in inoperable patients.

Genetic polymorphism within the promoter region might influence the promoter activity mediated by altering the binding efficiency of transcription factors. We demonstrated the promoter activity with the unfavorable allele C of SOCS5:rs3814039 was reduced compared to that with G allele (Fig. 2A-C). We analyzed the putative transcription factor binding sites in SOCS5:rs3814039_C and SOCS5:rs3814039_G by PROMO 44. Loss of putative p53 binding site was noted once the sequence of SOCS5 promoter change from G to C at rs3814039. Whether rs3814039 modulates the binding efficiency of p53 need further investigation. SOCS5:rs3768720 is within the regulatory region of 3'UTR which could influence RNA stability, translation efficiency, or microRNA binding efficiency. We also observed unfavorable SOCS5:rs3768720_C displayed reduced reporter expression compared to SOCS5:rs3768720_A (Fig. 2D-F). However, we did not found any obvious difference of human microRNA targeting between SOCS5:rs3768720_A and SOCS5:rs3768720_C from the online prediction database. We suggest rs3768720 SNP might effect on SOCS5 expression mediated by modulating mRNA transcript stability or translation efficiency.

SOCS5 is a potential negative regulator of EGFR, as has been demonstrated in both human cancer cells 25-26 and a Drosophila epithelial transformation model 45. A study further reported that SOCS5 improved control of influenza infection by inhibiting EGFR signaling 46. Our study provides evidence that SOCS5 might suppress EGFR expression in clinical ESCC tissue samples (Fig. 3A). In fact, we have also observed such a negative correlation in thymic tumor tissue (data not shown). We have demonstrated the ability of transiently over-expressing SOCS5 in regulating endogenous EGFR in 293T cells. The expression of SOCS5 was inversely correlated with the expression of EGFR, HER2 and phospho-AXL in Het-1A transformed esophageal cells. We also demonstrated the ability of SOCS5 to regulate ESCC cell migration by wound-healing assay (Fig. 5). Meanwhile, the 3'-UTR SNP rs3768720 is significantly correlated with distant metastasis of ESCC (Table 4), which reveal the possible regulatory function of SOCS5 in the metastasis of ESCC. EGFR has been found associated with postoperative recurrence 47, and poor prognosis of ESCC 48-49. We previously demonstrated that expression of AXL and/or HER2 significantly correlated with increased risk of distant metastasis 50. The results revealed that SOCS5 might regulate cell migration partly mediated by modulating the expressions of these metastasis-related receptor tyrosine kinases.

SOCS1, SOCS2, and SOCS3, the feedback regulators, have been demonstrated to turnover rapidly with a half-life of around 1 to 2 hours in COS-7 cells 42. Proteasome inhibitors have been shown to significantly stabilize SOCSs proteins 41, 51. In a neuroimmune study, rapid degradation of SOCS3 by a proteasome-dependent pathway allowed the corticotroph to go back to its basal state and thus be activated once again 52. Our study also found that proteasome inhibitor markedly increased the expression level of SOCS5 in ESCC cells (Fig. 4D). Consistently high expression might lead to inefficient function of SOCS5, which could partially explain why SOCS5 was frequently expression in ESCC tissues (Fig. 3A).

The prognostic implications of SOCS proteins on cancers are controversial. Higher expression levels of SOCS1, SOCS3, SOCS4, and SOCS7 have all been reported to associate with better prognosis in human breast cancer 53. In colorectal cancer (CRC), SOCS2 is also considered favorable for clinical outcome 54. SOCS1 was demonstrated to be unfavorable for clinical outcome in patients with human melanoma and in those with acute myeloid leukemia 55-56. The prognostic role of SOCS5 is hardly understood in cancers. Recently, SOCS5 has been reported over-expressed in hepatocellular carcinoma (HCC) tissues, and was correlated with poor prognosis of HCC patients 57.

Our results reveal a difference in the clinical impact of SOCS5 expression in adjacent normal tissue compared to esophageal tumor tissue. Strong expression of SOCS5 in normal tissue was correlated with better PFS though without reaching statistically significance, but the opposite trend was observed in tumor tissue (P=0.070 and 0.011 respectively, Fig. 3B). It is reasonable to speculate that SOCS5 expression in normal tissue plays an anti-tumor role which is mediated by regulating the oncogenic effects exerted by certain cytokines and growth factors to prevent disease progression. The question remains as to why an opposite relationship exists in tumor tissue regarding the correlation between SOCS5 expression and PFS? One possibility is that persistent expression of cytokines and growth factors in the tumor microenvironment might stimulate aberrant over-expression of SOCS5, and thus, the correlation of SOCS5 and unfavorable prognosis was observed.

In conclusion, our study is the first to demonstrate the genetic and molecular function of SOCS5 in ESCC prognosis. The hereditary SNPs of SOCS5 served as novel biomarkers for improving the prognosis of ESCC.

## Supplementary Material

Supplementary figures and tables.

## Figures and Tables

**Table 1:** Demographic and clinical characteristics of ESCC patients by survival status

Variables	Total	Training group N=268			Replication group N=364			Combined group N= 632		
		dead (N=195)	alive (N=73)	*p*-value	dead (N=260)	alive (N=104)	*p*-value	dead (N=455)	alive (N=177)	*p*-value
**Age (years)**										
<50	142 (22.5)	37(60.7)	24 (39.3)	0.029	59 (72.8)	22 (27.2)	0.276	96 (67.6)	46 (32.4)	0.087
50-65	297 (47.0)	102 (73.9)	36 (26.1)		107 (67.3)	52 (32.7)		209 (70.4)	88 (29.6)	
>65	193 (30.5)	56 (81.2)	13 (18.8)		94 (75.8)	30 (24.2)		150 (77.7)	43 (22.3)	
**Gender**										
Male	583 (92.2)	180(73.8)	64 (26.2)	0.237	245 (72.3)	94 (27.7)	0.190	425 (72.9)	158 (27.1)	0.080
Female	49 (7.8)	15 (62.5)	9 (37.5)		15 (60.0)	10 (40.0)		30 (61.2)	19 (38.8)	
**Stage**										
0	66 (10.4)	17 (58.6)	12 (41.4)		9 (24.3)	28 (75.7)		26 (39.4)	40 (60.6)	
I	124 (19.6)	27 (45.8)	32(54.2)	<0.001	35 (53.8)	30 (46.2)	<0.001	62 (50.0)	62 (50.0)	<0.001
II	155 (24.5)	48 (71.6)	19 (28.4)		59 (67.0)	29 (33.0)		107 (69.0)	48 (31.0)	
III	217 (34.3)	78 (89.7)	9 (10.3)		115 (88.5)	15 (11.5)		193 (88.9)	24 (11.1)	
IV	70 (11.1)	25 (96.2)	1 (3.8)		42 (95.5)	2 (4.5)		67 (95.7)	3 (4.3)	
**T-stage**										
0	86 (13.6)	26 (65.0)	14 (35.0)	<0.001	18 (39.1)	28 (60.9)	<0.001	44 (51.2)	42 (48.8)	<0.001
1	122 (19.3)	26 (45.6)	31 (54.4)		35 (53.8)	30 (46.2)		61 (50.0)	61 (50.0)	
2	122 (19.3)	37 (71.2)	15 (28.8)		49 (70.0)	21 (30.0)		86 (70.5)	36 (29.5)	
3	228 (36.1)	78 (87.6)	11 (12.4)		115 (82.7)	24 (17.3)		193 (84.6)	35 (15.4)	
4	74 (11.7)	28 (93.3)	2 (6.7)		43 (97.7)	1 (2.3)		71 (95.9)	3 (4.1)	
**N-stage**										
0	304 (48.1)	82 (60.3)	54 (39.7)	<0.001	92 (54.8)	76 (45.2)	<0.001	174 (57.2)	130 (42.8)	<0.001
1	288 (45.6)	110 (86.6)	17 (13.4)		141 (87.6)	20 (12.4)		251 (87.2)	37 (12.8)	
2	30 (4.7)	3 (60.0)	2 (40.0)		18 (72.0)	7 (28.0)		21 (70.0)	9 (30.0)	
3	10 (1.6)	0 (0)	0 (0)		9 (90.0)	1 (10.0)		9 (90.0)	1 (10.0)	
**M-stage**										
0	562 (88.9)	170 (70.2)	72 (29.8)	0.004	218 (68.1)	102 (31.9)	<0.001	388 (69.0)	174 (31.0)	<0.001
1	70 (11.1)	25 (96.2)	1 (3.8)		42 (95.5)	2 (4.5)		67 (95.7)	3 (4.3)	
**Tumor location**										
Upper	142 (22.5)	45 (84.9)	8 (15.1)	0.076	67 (75.3)	22 (24.7)	0.332	112 (78.9)	30 (21.1)	0.058
Middle	309 (48.9)	95 (70.9)	39 (29.1)		127 (72.6)	48 (27.4)		222 (71.8)	87 (28.2)	
Lower	181 (28.6)	55 (67.9)	26 (32.1)		66 (66.0)	34 (34.0)		121 (66.9)	60 (33.1)	
**Esophagectomy**										
No	226 (35.8)	89 (89.9)	10 (10.1)	<0.001	111 (87.4)	16 (12.6)	<0.001	200 (88.5)	26 (11.5)	<0.001
Yes	406 (64.2)	106 (62.7)	63 (37.3)		149 (62.9)	88 (37.1)		255 (62.8)	151 (37.2)	
**CCRT**										
No	156 (24.7)	42 (58.3)	30 (41.7)	0.003	58 (69.0)	26 (31.0)	0.306	100 (64.1)	56 (35.9)	0.006
Yes	446 (70.6)	142 (77.6)	41 (22.4)		186 (70.7)	77 (29.3)		328 (73.5)	118 (26.5)	
CT	16 (2.5)	7 (100.0)	0 (0)		9 (100.0)	0 (0)		16 (100.0)	0 (0)	
RT	11 (1.7)	11 (100)	1 (20.0)		5 (83.3)	1 (16.7)		9 (81.8)	2 (18.2)	
CT + RT	3 (0.5)	0 (0)	1 (100)		2 (100.0)	0 (0)		2 (66.7)	1 (33.3)	

**Table 2:** Association under multivariate analysis of SOCS SNPs with overall survival (OS) in ESCC patients under multivariate analysis

Genes	SNPs	Genotype	Training group (N= 268)			Replication group (N=364)			Combine group (N=632)		
			N	OS	*P-value	N	OS	*P-value	N	OS	*P-value
SOCS1	rs33932899	GG	158	1		200	1		358	1	
		CG	100	0.93 (0.69-1.27)	0.668	140	1.16 (0.89-1.52)	0.287	240	1.04 (0.85-1.27)	0.703
		CC	9	0.61 (0.27-1.35)	0.220	24	1.20 (0.74-1.93)	0.465	33	0.85 (0.63-1.42)	0.799
	rs1559392	CC	157	1							
		CT	100	0.95 (0.70-1.30)	0.755						
		TT	10	0.71 (0.33-1.51)	0.376						
	rs243324	CC	157	1		190	1		347	1	
		CT	99	0.92 (0.68-1.26)	0.604	147	1.11 (0.85-1.46)	0.432	246	1.01 (0.83-1.24)	0.899
		TT	12	0.84 (0.41-1.72)	0.639	27	1.21 (0.76-1.92)	0.416	39	1.06 (0.73-1.55)	0.762
	rs243327	CC	157	1							
		TC	98	0.92 (0.68-1.26)	0.617						
		TT	12	0.90 (0.45-1.79)	0.769						
	rs243330	AA	158								
		AG	98	0.95 (0.70-1.29)	0.738						
		GG	11	0.80 (0.39-1.65)	0.552						
SOCS3	rs4969169	CC	121	1							
		CT	127	0.95 (0.70-1.29)	0.740						
		TT	20	0.79 (0.44-1.43)	0.441						
	rs9892622	AA	100	1							
		AG	124	1.08(0.78-1.48)	0.654						
		GG	44	1.35 (0.88-2.08)	0.172						
	rs2280148	AA	148	1		199	1		347	1	
		AC	105	0.90 (0.66-1.22)	0.489	139	1.19 (0.91-1.55)	0.209	244	1.04 (0.86-1.27)	0.681
		CC	14	0.66 (0.32-1.34)	0.247	26	1.03 (0.62-1.71)	0.918	40	0.89 (0.59-1.33)	0.562
SOCS5	rs3814039	GG	62	1		69	1		131	1	
		GC	126	0.96 (0.66-1.40)	0.831	186	1.15 (0.81-1.63)	0.437	312	1.07 (0.84-1.38)	0.583
		CC	78	1.40 (0.94-2.09)	0.098	108	1.33 (0.92-1.92)	0.127	186	1.34 (1.03-1.74)	**0.032**
		***Trend***		1.20 (0.98-1.48)	0.081		1.15 (0.97-1.38)	0.116		1.17 (1.02-1.33)	**0.023**
	rs3738890	CC	67	1		68	1		135	1	
		GC	121	1.11 (0.77-1.61)	0.577	186	1.17 (0.83-1.66)	0.366	307	1.15 (0.90-1.47)	0.266
		GG	79	1.49 (1.00-2.21)	**0.046**	107	1.34 (0.93-1.93)	0.119	186	1.38 (1.06-1.80)	**0.016**
		***Trend***		1.23 (1.01-1.50)	**0.042**		1.15 (0.97-1.38)	0.114		1.18 (1.03-1.34)	**0.014**
	rs6738426	AA	217	1							
		AG	50	1.11 (0.76-1.60)	0.593						
	rs3768720	AA	64	1		75	1		139	1	
		CA	133	1.12 (0.77-1.63)	0.553	190	1.12 (0.80-1.57)	0.498	323	1.13 (0.89-1.45)	0.325
		CC	71	1.67 (1.11-2.51)	**0.014**	99	1.37 (0.95-1.96)	0.092	170	1.46 (1.12-1.91)	**0.005**
		***Trend***		1.31 (1.06-1.61)	**0.012**		1.17 (0.98-1.41)	0.081		1.22 (1.06-1.39)	**0.004**
	rs12051836	TT	174	1							
		TC	84	0.98 (0.71-1.34)	0.896						
		CC	10	0.87 (0.38-2.01)	0.743						
CISH	rs414171	TT	163	1							
		AA	104	1.13 (0.84-1.51)	0.435						
	rs2239751	AA	132	1		150	1		282	1	
		AC	104	0.79 (0.57-1.09)	0.145	164	0.92 (0.70-1.21)	0.563	268	0.91 (0.74-1.11)	0.349
		CC	32	1.06 (0.65-1.75)	0.811	50	0.79 (0.53-1.19)	0.261	82	0.89 (0.66-1.22)	0.472
	rs622502	CC	226	1		322	1		548	1	
		CT	41	0.95 (0.61-1.47)	0.806	40	1.00 (0.68-1.48)	0.947	81	0.95 (0.71-1.27)	0.736
		TT	1	18.23 (2.29-145.35)	**0.006**	2	0.38 (0.09-1.59)	0.183	3	0.60 (0.19-1.93)	0.393

Adjusted for age, gender, stage, surgical status and CCRT.

**Table 3:** Association under multivariate analysis of SOCS SNPs with progression-free overall survival (PFS) in ESCC patients

Genes	SNPs	genotype	Training group (N= 268)			Replication group (N=364)			Combine group (N=632)		
			N	PFS	*P-value	N	PFS	*P-value	N	PFS	*P-value
SOCS1	rs33932899	GG	158	1		200	1		358	1	
		CG	100	1.06 (0.80-1.41)	0.698	140	1.10 (0.86-1.42)	0.449	240	1.08 (0.89-1.30)	0.436
		CC	9	0.49 (0.22-1.09)	0.080	24	0.93 (0.58-1.48)	0.756	33	0.80 (0.54-1.18)	0.260
	rs28503542	GG	267	-	-	-	-	-	-	-	-
	rs1559392	CC	157	1							
		CT	100	1.05 (0.79-1.40)	0.747						
		TT	10	0.57 (0.26-1.21)	0.142						
	rs243324	CC	157	1		190	1		347	1	
		CT	99	1.03 (0.78-1.38)	0.823	147	1.01 (0.79-1.30)	0.942	246	1.02 (0.84-1.23)	0.857
		TT	12	0.60 (0.29-1.23)	0.161	27	0.93 (0.59-1.45)	0.743	39	0.84 (0.58-1.21)	0.345
	rs243327	CC	157	1							
		TC	98	1.03 (0.77-1.37)	0.858						
		TT	12	0.70 (0.35-1.40)	0.315						
	rs243330	AA	158	1							
		AG	98	1.06 (0.80-1.41)	0.695						
		GG	11	0.63 (0.31-1.29)	0.207						
SOCS3	rs4969169	CC	121	1							
		CT	127	0.99 (0.75-1.32)	0.958						
		TT	20	0.74 (0.42-1.29)	0.286						
	rs9892622	AA	100	1							
		AG	124	0.97 (0.71-1.31)	0.827						
		GG	44	1.04 (0.69-1.57)	0.835						
	rs2280148	AA	148	1		199	1		347	1	
		AC	105	1.28 (0.95-1.73)	0.106	139	1.19 (0.92-1.53)	0.181	244	1.18 (0.98-1.43)	0.082
		CC	14	0.79 (0.39-1.58)	0.498	26	1.04 (0.65-1.67)	0.870	40	0.96 (0.65-1.40)	0.812
SOCS5	rs3814039	GG	62	1		69	1		131	1	
		GC	126	0.95 (0.67-1.35)	0.770	186	1.25 (0.90-1.74)	0.191	312	1.10 (0.87-1.39)	0.420
		CC	78	1.44 (0.99-2.10)	0.057	108	1.45 (1.03-2.06)	**0.034**	186	1.40 (1.09-1.79)	**0.009**
		***Trend***		1.22 (1.01-1.49)	**0.045**		1.20 (1.01-1.42)	**0.034**		1.19 (1.05-1.35)	**0.007**
	rs3738890	CC	67	1		68	1		135	1	
		GC	121	1.13 (0.80-1.61)	0.479	186	1.27 (0.92-1.76)	0.148	307	1.20 (0.95-1.52)	0.118
		GG	79	1.52 (1.04-2.20)	**0.029**	107	1.41 (1.00-2.00)	0.051	186	1.41 (1.09-1.81)	**0.008**
		***Trend***		1.24 (1.03-1.50)	**0.027**		1.18 (1.00-1.40)	0.055		1.18 (1.05-1.34)	**0.008**
	rs6738426	AA	217	1							
		AG	50	1.30 (0.91-1.85)	0.151						
	rs3768720	AA	64	1		75	1		139	1	
		CA	133	1.23 (0.87-1.75)	0.244	190	1.25 (0.91-1.71)	0.164	323	1.25 (0.99-1.57)	0.061
		CC	71	1.61 (1.09-2.37)	**0.017**	99	1.53 (1.08-2.16)	**0.016**	170	1.51 (1.17-1.95)	**0.002**
		***Trend***		1.27 (1.05-1.54)	**0.016**		1.24 (1.04-1.47)	**0.015**		1.23 (1.08-1.39)	**0.002**
	rs12051836	TT	174	1							
		TC	84	0.94 (0.70-1.28)	0.701						
		CC	10	1.26 (0.60-2.64)	0.540						
CISH	rs414171	TT	163	1							
		AA	104	0.97 (0.73-1.28)	0.813						
	rs2239751	AA	132	1		150	1		282	1	
		AC	104	0.92 (0.68-1.24)	0.568	164	1.05 (0.81-1.37)	0.708	268	1.04 (0.86-1.25)	0.721
		CC	32	1.15 (0.72-1.82)	0.560	50	0.97 (0.66-1.43)	0.873	82	1.02 (0.77-1.37)	0.873
	rs622502	CC	226	1		322	1		548	1	
		CT	41	1.21 (0.80-1.82)	0.374	40	0.84 (0.57-1.21)	0.343	81	0.96 (0.73-1.26)	0.758
		TT	1	8.70 (1.14-66.73)	**0.037**	2	1.44 (0.34-6.13)	0.619	3	2.03 (0.63-6.53)	0.233

*Adjusted for age, gender, stage, surgical status and CCRT.

**Table 4:** Association under multivariate analysis of SOCS SNPs with local recurrence and distant metastasis in ESCC patients

Variable	Distant metastasis (No:Yes=153:216)			Local recurrence (No:Yes=187:68)		
	N	OR (95% CI)	*P-value	N	OR (95% CI)	*P-value
**rs3814039**						
GG	79	1		59	1	
GC	193	1.67 (0.91-3.05)	0.100	132	0.79 (0.37-1.67)	0.536
CC	95	1.71 (0.86-3.42)	0.128	62	1.31 (0.57-3.02)	0.525
***Trend***		1.30 (0.92-1.83)	0.143		1.16 (0.75-1.78)	0.504
**rs3738890**						
CC	83	1		63	1	
GC	190	0.64 (0.33-1.27)	0.205	127	0.92 (0.44-1.92)	0.814
GG	93	1.25 (0.70-2.23)	0.447	63	1.43 (0.63-3.27)	0.391
***Trend***		0.81 (0.57-1.14)	0.225		1.53 (0.67-3.53)	0.316
**rs3768720**						
AA	86	1		66	1	
CA	198	1.96 (1.09-3.52)	**0.025**	132	0.86 (0.42-1.77)	0.689
CC	85	2.17 (1.08-4.40)	**0.030**	57	1.20 (0.52-2.77)	0.670
***Trend***		1.49 (1.04-2.12)	**0.028**		1.09 (0.71-1.66)	0.706

*Adjusted for age, gender, stage, surgical status and CCRT.

**Table 5:** Correlation of SOCS5 with distant metastasis of ESCC

Variables	Distant metastasis (N=60)		
	No	Yes	*p*-value
**Normal_tissue SOCS5**			0.004
Weak	10 (41.7)	14 (58.3)	
Strong	28 (77.8)	8 (22.2)	
**Tumor_tissue SOCS5**			0.155
Weak	14 (77.8)	4 (22.2)	
Strong	24 (57.1)	18 (42.9)	

## References

[1] Islami F, Fedirko V, Tramacere I, Bagnardi V, Jenab M, Scotti L (2011). Alcohol drinking and esophageal squamous cell carcinoma with focus on light-drinkers and never-smokers: a systematic review and meta-analysis. Int J Cancer.

[2] Tran GD, Sun XD, Abnet CC, Fan JH, Dawsey SM, Dong ZW (2005). Prospective study of risk factors for esophageal and gastric cancers in the Linxian general population trial cohort in China. Int J Cancer.

[3] Miyashita M, Tajiri T, Sasajima K, Makino H, Maruyama H, Nomura T (2003). Response to preoperative chemotherapy affects prognosis in esophageal cancer. Dis Esophagus.

[4] Sugimachi K, Inokuchi K, Kuwano H, Kai H, Okamura T, Okudaira Y (1983). Patterns of Recurrence after Curative Resection for Carcinoma of the Thoracic Part of the Esophagus. Surg Gynecol Obstet.

[5] Nakagawa S, Kanda T, Kosugi SI, Ohashi M, Suzuki T, Hatakeyama K (2004). Recurrence pattern of squamous cell carcinoma of the thoracic esophagus after extended radical esophagectomy with three-field lymphadenectomy. J Am Coll Surgeons.

[6] Su XD, Zhang DK, Zhang X, Lin P, Long H, Rong TH (2014). Prognostic factors in patients with recurrence after complete resection of esophageal squamous cell carcinoma. J Thorac Dis.

[7] Allum WH, Stenning SP, Bancewicz J, Clark PI, Langley RE (2009). Long-term results of a randomized trial of surgery with or without preoperative chemotherapy in esophageal cancer. J Clin Oncol.

[8] Diakowska D (2013). Cytokines Association with Clinical and Pathological Changes in Esophageal Squamous Cell Carcinoma. Dis Markers.

[9] Lukaszewicz-Zajac M, Mroczko B, Kozlowski M, Niklinski J, Laudanski J, Szmitkowski M (2011). Higher importance of interleukin 6 than classic tumor markers (carcinoembryonic antigen and squamous cell cancer antigen) in the diagnosis of esophageal cancer patients. Dis Esophagus.

[10] Oka M, Yamamoto K, Takahashi M, Hakozaki M, Abe T, Iizuka N (1996). Relationship between serum levels of interleukin 6, various disease parameters and malnutrition in patients with esophageal squamous cell carcinoma. Cancer Res.

[11] Leu CM, Wong FH, Chang C, Huang SF, Hu CP (2003). Interleukin-6 acts as an antiapoptotic factor in human esophageal carcinoma cells through the activation of both STAT3 and mitogen-activated protein kinase pathways. Oncogene.

[12] Chen MF, Lu MS, Chen PT, Chen WC, Lin PY, Lee KD (2012). Role of interleukin 1 beta in esophageal squamous cell carcinoma. J Mol Med.

[13] Krzystek-Korpacka M, Matusiewicz M, Diakowska D, Grabowski K, Blachut K, Kortieuny D (2008). Elevation of circulating interleukin-8 is related to lymph node and distant metastases in esophageal squamous cell carcinomas - Implication for clinical evaluation of cancer patient. Cytokine.

[14] Diakowska D, Markocka-Maczka K, Grabowski K, Lewandowski A (2006). Serum interleukin-12 and interleukin-18 levels in patients with oesophageal squamous cell carcinoma. Exp Oncol.

[15] Ren Y, Cao B, Law S, Xie Y, Lee PY, Cheung L (2005). Hepatocyte growth factor promotes cancer cell migration and angiogenic factors expression: a prognostic marker of human esophageal squamous cell carcinomas. Clin Cancer Res.

[16] Imsumran A, Adachi Y, Yamamoto H, Li R, Wang Y, Min YF (2007). Insulin-like growth factor-I receptor as a marker for prognosis and a therapeutic target in human esophageal squamous cell carcinoma. Carcinogenesis.

[17] Yang PW, Hsieh MS, Huang YC, Hsieh CY, Chiang TH, Lee JM (2014). Genetic variants of EGF and VEGF predict prognosis of patients with advanced esophageal squamous cell carcinoma. Plos One.

[18] Trengove MC, Ward AC (2013). SOCS proteins in development and disease. Am J Clin Exp Immunol.

[19] Yoshimura A, Naka T, Kubo M (2007). SOCS proteins, cytokine signalling and immune regulation. Nat Rev Immunol.

[20] Tischoff I, Hengge UR, Vieth M, Ell C, Stolte M, Weber A (2007). Methylation of SOCS-3 and SOCS-1 in the carcinogenesis of Barrett's adenocarcinoma. Gut.

[21] Hussain S, Singh N, Salam I, Bandil K, Yuvaraj M, Akbar Bhat M (2011). Methylation-mediated gene silencing of suppressor of cytokine signaling-1 (SOCS-1) gene in esophageal squamous cell carcinoma patients of Kashmir valley. J Recept Signal Transduct Res.

[22] Sugase T, Takahashi T, Serada S, Nakatsuka R, Fujimoto M, Ohkawara T (2017). Suppressor of cytokine signaling-1 gene therapy induces potent antitumor effect in patient-derived esophageal squamous cell carcinoma xenograft mice. Int J Cancer.

[23] Hartavi M, Kurt E, Oral B, Olmez OF, Cubukcu E, Deligonul A (2013). The SOCS-1-1478CA/del Polymorphism is not Associated with Colorectal Cancer or Age at Onset in Turkish Subjects. Asian Pac J Cancer P.

[24] Igci M, Cakmak EA, Oztuzcu S, Bayram A, Arslan A, Gogebakan B (2012). Mutational Screening of the SOCS3 Gene Promoter in Metastatic Colorectal Cancer Patients. Genet Test Mol Bioma.

[25] Nicholson SE, Metcalf D, Sprigg NS, Columbus R, Walker F, Silva A (2005). Suppressor of cytokine signaling (SOCS)-5 is a potential negative regulator of epidermal growth factor signaling. P Natl Acad Sci USA.

[26] Kario E, Marmor MD, Adamsky K, Citri A, Amit I, Amariglio N (2005). Suppressors of cytokine signaling 4 and 5 regulate epidermal growth factor receptor signaling. J Biol Chem.

[27] Ni R, Ihara K, Miyako K, Takemoto M, Ishimura M, Kohno H (2006). Association study of polymorphisms in SOCS family genes with type 1 diabetes mellitus. Int J Immunogenet.

[28] Yang PW, Hsieh MS, Chang YH, Huang PM, Lee JM (2017). Genetic polymorphisms of ATG5 predict survival and recurrence in patients with early-stage esophageal squamous cell carcinoma. Oncotarget.

[29] Hu CP, Hsieh HG, Chien KY, Wang PY, Wang CI, Chen CY (1984). Biologic properties of three newly established human esophageal carcinoma cell lines. J Natl Cancer Inst.

[30] Wuu KD, Cheng MY, Wang-Wuu S, Hu CP, Chang CM (1986). Chromosome analysis on a cell line (CE48T/VGH) derived from a human esophageal carcinoma. Cancer Genet Cytogenet.

[31] Shimada Y, Imamura M, Wagata T, Yamaguchi N, Tobe T (1992). Characterization of 21 Newly Established Esophageal Cancer Cell-Lines. Cancer.

[32] Stoner GD, Kaighn ME, Reddel RR, Resau JH, Bowman D, Naito Z (1991). Establishment and characterization of SV40 T-antigen immortalized human esophageal epithelial cells. Cancer Res.

[33] Chua HH, Lee HH, Chang SS, Lu CC, Yeh TH, Hsu TY (2007). Role of the TSG101 gene in Epstein-Barr virus late gene transcription. J Virol.

[34] Yang PW, Hung MC, Hsieh CY, Tung EC, Wang YH, Tsai JC (2013). The effects of Photofrin-mediated photodynamic therapy on the modulation of EGFR in esophageal squamous cell carcinoma cells. Lasers Med Sci.

[35] Hsu SC, Hung MC (2007). Characterization of a novel tripartite nuclear localization sequence in the EGFR family. J Biol Chem.

[36] Yang PW, Liu YC, Chang YH, Lin CC, Huang PM, Hua KT (2019). Cabozantinib (XL184) and R428 (BGB324) Inhibit the Growth of Esophageal Squamous Cell Carcinoma (ESCC). Frontiers in Oncology.

[37] Tao YM, Ma C, Fan QH, Wang YN, Han T, Sun CX (2018). MicroRNA-1296 Facilitates Proliferation, Migration And Invasion Of Colorectal Cancer Cells By Targeting SFPQ. J Cancer.

[38] Mostecki J, Cassel SL, Klimecki WT, Stern DA, Knisz J, Iwashita S (2011). A SOCS-1 Promoter Variant Is Associated with Total Serum IgE Levels. J Immunol.

[39] Jamshidi Y, Snieder H, Wang X, Spector TD, Carter ND, O'Dell SD (2006). Common polymorphisms in SOCS3 are not associated with body weight, insulin sensitivity or lipid profile in normal female twins. Diabetologia.

[40] Harada M, Nakashima K, Hirota T, Shimizu M, Doi S, Fujita K (2007). Functional polymorphism in the suppressor of cytokine signaling 1 gene associated with adult asthma. Am J Respir Cell Mol Biol.

[41] Zhang JG, Farley A, Nicholson SE, Willson TA, Zugaro LM, Simpson RJ (1999). The conserved SOCS box motif in suppressors of cytokine signaling binds to elongins B and C and may couple bound proteins to proteasomal degradation. P Natl Acad Sci USA.

[42] Siewert E, Muller-Esterl W, Starr R, Heinrich PC, Schaper F (1999). Different protein turnover of interleukin-6-type cytokine signalling components. European Journal of Biochemistry.

[43] Guillem V, Amat P, Cervantes F, Alvarez-Larran A, Cervera J, Maffioli M (2012). Functional polymorphisms in SOCS1 and PTPN22 genes correlate with the response to imatinib treatment in newly diagnosed chronic-phase chronic myeloid leukemia. Leuk Res.

[44] Farre D, Roset R, Huerta M, Adsuara JE, Rosello L, Alba MM (2003). Identification of patterns in biological sequences at the ALGGEN server: PROMO and MALGEN. Nucleic Acids Res.

[45] Herranz H, Hong X, Hung NT, Voorhoeve PM, Cohen SM (2012). Oncogenic cooperation between SOCS family proteins and EGFR identified using a Drosophila epithelial transformation model. Gene Dev.

[46] Kedzierski L, Tate MD, Hsu AC, Kolesnik TB, Linossi EM, Dagley L (2017). Suppressor of cytokine signaling (SOCS)5 ameliorates influenza infection via inhibition of EGFR signaling. Elife.

[47] Yamamoto Y, Yamai H, Seike J, Yoshida T, Takechi H, Furukita Y (2012). Prognosis of Esophageal Squamous Cell Carcinoma in Patients Positive for Human Epidermal Growth Factor Receptor Family Can Be Improved by Initial Chemotherapy with Docetaxel, Fluorouracil, and Cisplatin. Annals of Surgical Oncology.

[48] Gibault L, Metges JP, Conan-Charlet V, Lozac'h P, Robaszkiewicz M, Bessaguet C (2005). Diffuse EGFR staining is associated with reduced overall survival in locally advanced oesophageal squamous cell cancer. Brit J Cancer.

[49] Jiang DX, Li XJ, Wang HX, Shi Y, Xu C, Lu SH (2015). The prognostic value of EGFR overexpression and amplification in Esophageal squamous cell Carcinoma. Bmc Cancer.

[50] Hsieh MS, Yang PW, Wong LF, Lee JM (2016). The AXL receptor tyrosine kinase is associated with adverse prognosis and distant metastasis in esophageal squamous cell carcinoma. Oncotarget.

[51] Auernhammer CJ, Melmed S (2001). The central role of SOCS-3 in integrating the neuro-immunoendocrine interface. Journal of Clinical Investigation.

[52] Bousquet C, Susini C, Melmed S (1999). Inhibitory roles for SHP-1 and SOCS-3 following pituitary proopiomelanocortin induction by leukemia inhibitory factor. Journal of Clinical Investigation.

[53] Sasi W, Jiang WG, Sharma A, Mokbel K (2010). Higher expression levels of SOCS 1,3,4,7 are associated with earlier tumour stage and better clinical outcome in human breast cancer. Bmc Cancer.

[54] Letellier E, Schmitz M, Baig K, Beaume N, Schwartz C, Frasquilho S (2014). Identification of SOCS2 and SOCS6 as biomarkers in human colorectal cancer. Brit J Cancer.

[55] Hou HA, Lu JW, Lin TY, Tsai CH, Chou WC, Lin CC (2017). Clinico-biological significance of suppressor of cytokine signaling 1 expression in acute myeloid leukemia. Blood Cancer J.

[56] Li Z, Metze D, Nashan D, Muller-Tidow C, Serve HL, Poremba C (2004). Expression of SOCS-1, suppressor of cytokine signalling-1, in human melanoma. J Invest Dermatol.

[57] Zhang M, Liu SH, Chua MS, Li HR, Luo DG, Wang S (2019). SOCS5 inhibition induces autophagy to impair metastasis in hepatocellular carcinoma cells via the PI3K/Akt/mTOR pathway. Cell Death & Disease.

[58] Butler TAJ, Paul JW, Chan EC, Smith R, Tolosa JM (2019). Misleading Westerns: Common Quantification Mistakes in Western Blot Densitometry and Proposed Corrective Measures. Biomed Res Int.

