# Supplementary material for: The genetic effect and molecular function of the SOCS5 in the prognosis of esophageal squamous cell carcinoma
Source: J Cancer. 2021 Feb 22;12(8):2216–29. doi: 10.7150/jca.51806 (PMC7974883; doi:10.7150/jca.51806)
Supplement: Supplementary file 1 — Supplementary figures and tables. [file jcav12p2216s1.pdf]

Supplementary materials

Supplementary figure legends.

Fig.S1. Kaplan-Meier estimates of overall survival by the genotypes of SOCS5:rs3814039 (A and B), SOCS5: rs3738890 (C and D), and SOCS5: rs3768720 (E and F) in ESCC patients who received [op (+), B, D and F] or did not received surgical dissection [op (-), A, C and E]. MST: median survival time (months).

Fig.S2. Kaplan-Meier estimates of progression-free survival by the genotypes of SOCS5:rs3814039 (A and B), SOCS5: rs3738890 (C and D), and SOCS5: rs3768720 (E and F) in ESCC patients who received [op (+), B, D and F] or did not received surgical dissection [op (-), A, C and E]. MST: median survival time (months).

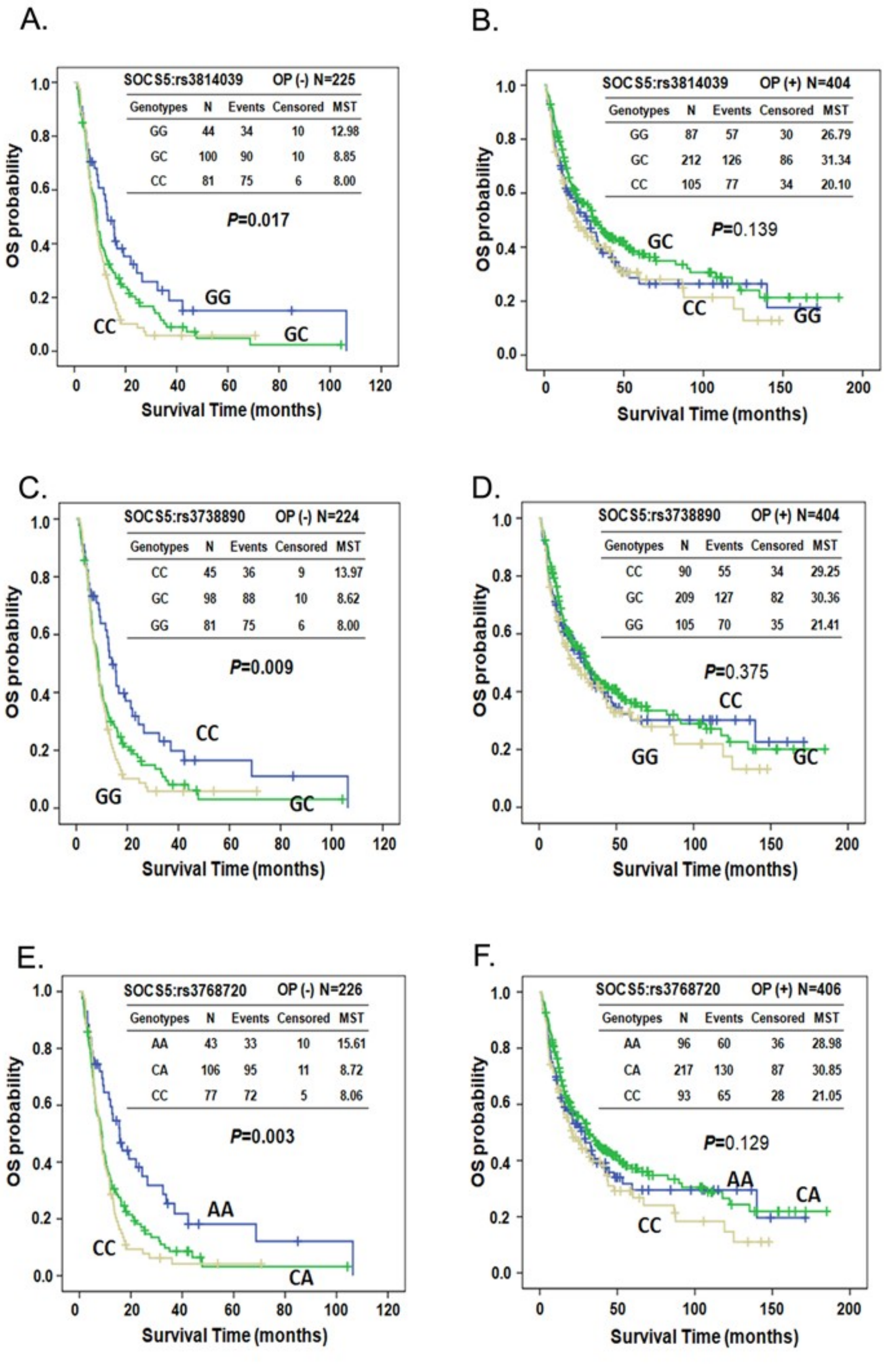

Fig.S1. Yang et al.,

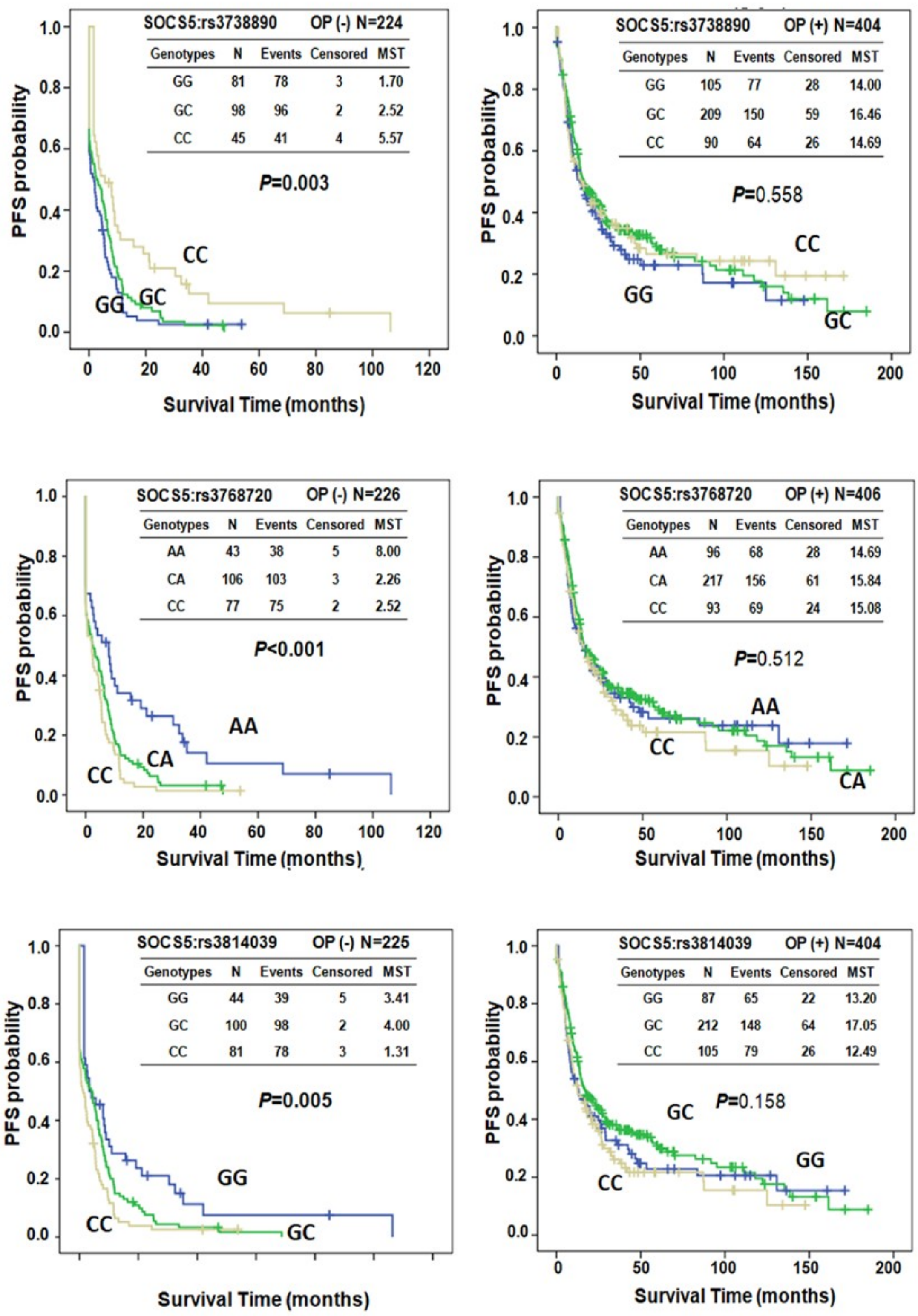
SOCS5:rs3738890 OP (-) N=224
Genotypes N Events Censored MST
GG 81 78 3 1.70
GC 98 96 2 2.52
CC 45 41 4 5.57
P=0.003
PFS probability
Survival Time (months)
SOCS5:rs3738890 OP (+) N=404
Genotypes N Events Censored MST
GG 105 77 28 14.00
GC 209 150 59 16.46
CC 90 64 26 14.69
P=0.558
SOCS5:rs3768720 OP (-) N=226
Genotypes N Events Censored MST
AA 43 38 5 8.00
CA 106 103 3 2.26
CC 77 75 2 2.52
P<0.001
SOCS5:rs3768720 OP (+) N=406
Genotypes N Events Censored MST
AA 96 68 28 14.69
CA 217 156 61 15.84
CC 93 69 24 15.08
P=0.512
SOCS5:rs3814039 OP (-) N=225
Genotypes N Events Censored MST
GG 44 39 5 3.41
GC 100 98 2 4.00
CC 81 78 3 1.31
P=0.005
SOCS5:rs3814039 OP (+) N=404
Genotypes N Events Censored MST
GG 87 65 22 13.20
GC 212 148 64 17.05
CC 105 79 26 12.49
P=0.158

Fig.S2

Table S1. Patient's characteristics by SOCS5 expression level

	Total	Normal tissues			Tumor tissues		
**SOCS5 Variables**		**Strong N=37 (59.7)**	**Weak N=25 (40.3)**	***p*-value**	**Strong N=44 (71.0)**	**Weak N=18 (29.0)**	***p*-value**
**Age (years)**							
<50	10 (16.1)	6 (60.0)	4 (40.0)	0.457	9 (90.0)	1 (10.0)	0.110
50-65	35 (56.5)	23 (65.7)	12 (34.3)		21 (60.0)	14 (40.0)	
>65	17 (27.4)	8 (47.1)	9 (52.9)		14 (82.4)	3 (17.6)	
**Gender**							
Male	60 (96.8)	35 (58.3)	25 (41.7)	0.511	42 (70.0)	18 (30.0)	1.000
Female	2 (3.2)	2 (100.0)	0 (0)		2 (100.0)	0 (0)	
**Stage**							
0+I	35 (56.5)	20 (57.1)	15 (42.9)		22 (62.9)	13 (37.1)	
II	18 (29.0)	10 (55.6)	8 (44.4)	0.553	16 (88.9)	2 (11.1)	0.146
III+IV	9 (14.5)	7 (77.8)	2 (22.2)		6 (66.7)	3 (33.3)	
**Tumor location**							
Upper	13 (21.0)	7 (53.8)	6 (46.2)	0.820	10 (76.9)	3 (23.1)	0.586
Middle	24 (38.7)	14 (58.3)	10 (41.7)		15 (62.5)	9 (37.5)	
Lower	25 (40.3)	16 (64.0)	9 (36.0)		19 (76.0)	6 (24.0)	
**CCRT**							
No	10 (16.1)	6 (60.0)	4 (40.0)	1.000	7 (70.0)	3 (30.0)	1.000
Yes	52 (83.9)	31 (59.6)	21 (40.4)		37 (71.2)	15 (28.8)	
